# Rapid fluorescence imaging of human hepatocellular carcinoma using the β-galactosidase-activatable fluorescence probe SPiDER-βGal

**DOI:** 10.1038/s41598-021-97073-1

**Published:** 2021-09-09

**Authors:** Soichiro Ogawa, Hidemasa Kubo, Yasutoshi Murayama, Takeshi Kubota, Masayuki Yubakami, Tatsuya Matsumoto, Yusuke Yamamoto, Ryo Morimura, Hisashi Ikoma, Kazuma Okamoto, Mako Kamiya, Yasuteru Urano, Eigo Otsuji

**Affiliations:** 1 Division of Digestive Surgery, Department of Surgery, Kyoto Prefectural University of Medicine, 465 Kajii-cho, Kamigyo-ku, Kyoto, 602-8566 Japan; 2 Graduate School of Medicine, The University of Tokyo, 7-3-1 Hongo, Bunkyo-ku, Tokyo, 113-0033 Japan; 3 Graduate School of Pharmaceutical Sciences, The University of Tokyo, 7-3-1 Hongo, Bunkyo-ku, Tokyo, 113-0033 Japan; 4 CREST (Japan) Agency for Medical Research and Development (AMED), 1-7-1 Otemachi, Chiyoda-ku, Tokyo, 100-0004 Japan

**Keywords:** Cancer, Chemical biology, Gastroenterology, Oncology

## Abstract

Fluorescence imaging of tumours facilitates rapid intraoperative diagnosis. Thus far, a promising activatable fluorescence probe for hepatocellular carcinoma (HCC) has not been developed. Herein, the utility of the fluorescence imaging of HCC using a β-galactosidase (β-Gal)-activatable fluorescence probe SPiDER-βGal was examined. β-Gal activity was measured in cryopreserved tissues from 68 patients. Live cell imaging of HCC cell lines and imaging of tumour-bearing model mice were performed using SPiDER-βGal. Furthermore, fluorescence imaging was performed in 27 freshly resected human HCC specimens. In cryopreserved samples, β-Gal activity was significantly higher in tumour tissues than in non-tumour tissues. Fluorescence was observed in HCC cell lines. In mouse models, tumours displayed stronger fluorescence than normal liver tissue. In freshly resected specimens, fluorescence intensity in the tumour was significantly higher than that in non-tumour liver specimens as early as 2 min after spraying. Receiver operating characteristic curves were generated to determine the diagnostic value of SPiDER-βGal 10 min after its spraying; an area under the curve of 0.864, sensitivity of 85.2%, and specificity of 74.1% were observed for SPiDER-βGal. SPiDER-βGal is useful for the rapid fluorescence imaging of HCC. Fluorescence imaging guided by SPiDER-βGal would help surgeons detect tumours rapidly and achieve complete liver resection.

## Introduction

Liver cancer, the sixth most frequently diagnosed cancer worldwide and the fourth most common cause of cancer-related mortality^1^, has a poor prognosis with a 5-year relative survival rate of 18.1%^2^. Hepatocellular carcinoma (HCC) accounts for most primary liver cancers. Surgical resection is one of the most effective treatments for HCC. However, > 70% of patients have tumour recurrence within 5 years after hepatectomy for HCC^3^. The presence of pathological cancer remnants after liver resection is a major recurrence risk^4^. Therefore, even minute amounts of residual cancerous tissues must be prevented in preserved livers. To achieve complete resection, it is important to identify the location of the tumour intraoperatively and confirm that no cancer is exposed at the resected surface of the liver. A modality helping surgeons detect HCC intraoperatively is therefore highly desirable.

Fluorescence-guided surgery has been developed as a safe and reliable surgical method^5^. Indocyanine green (ICG)^6–12^ and 5-aminolevulinic acid (5-ALA)^13–15^ are fluorescent molecules often used intraoperatively to facilitate the complete resection of HCC. Recently, activatable fluorescence probes, which are normally non-fluorescent but can be activated through cancer-specific enzymes, have been developed as novel fluorescent diagnostics that can specifically and rapidly image cancer after topical spraying. For example, a fluorescence probe targeting dipeptidylpeptidase IV has been used to detect oesophageal squamous cell carcinoma^16,17^ and adenocarcinoma of the oesophagogastric junction^18^. Similarly, a gamma-glutamyl transpeptidase (GGT)-activatable fluorescence probe can reportedly detect ovarian cancer^19^, breast cancer^20^, lung cancer^21^, liver cancer^22^, superficial head and neck squamous cell carcinoma^23^, and metastatic lymph nodes in colorectal cancer^24^. However, target enzymes and fluorescence probes that are sufficiently effective for use in HCC have not been determined. β-galactosidase (β-Gal)-targeted fluorescence probes can help visualise ovarian cancer cells, and the small peritoneal metastases from ovarian cancer in mice^25,26^. Recently, we reported that β-Gal is a target enzyme for detecting the peritoneal metastasis in gastric cancer^27^. However, the fluorescence imaging of HCC using β-Gal-targeted fluorescence probes has not been reported.

This study focused on β-Gal as a candidate enzyme for the fluorescence imaging of HCC and aimed to examine the feasibility of using the SPiDER-βGal, β-Gal-targeted fluorescence probe^28^, for the intraoperative rapid fluorescence imaging of HCC.

## Results

### β-Gal activity in cryopreserved human HCC tissue samples

**Figure 1:**
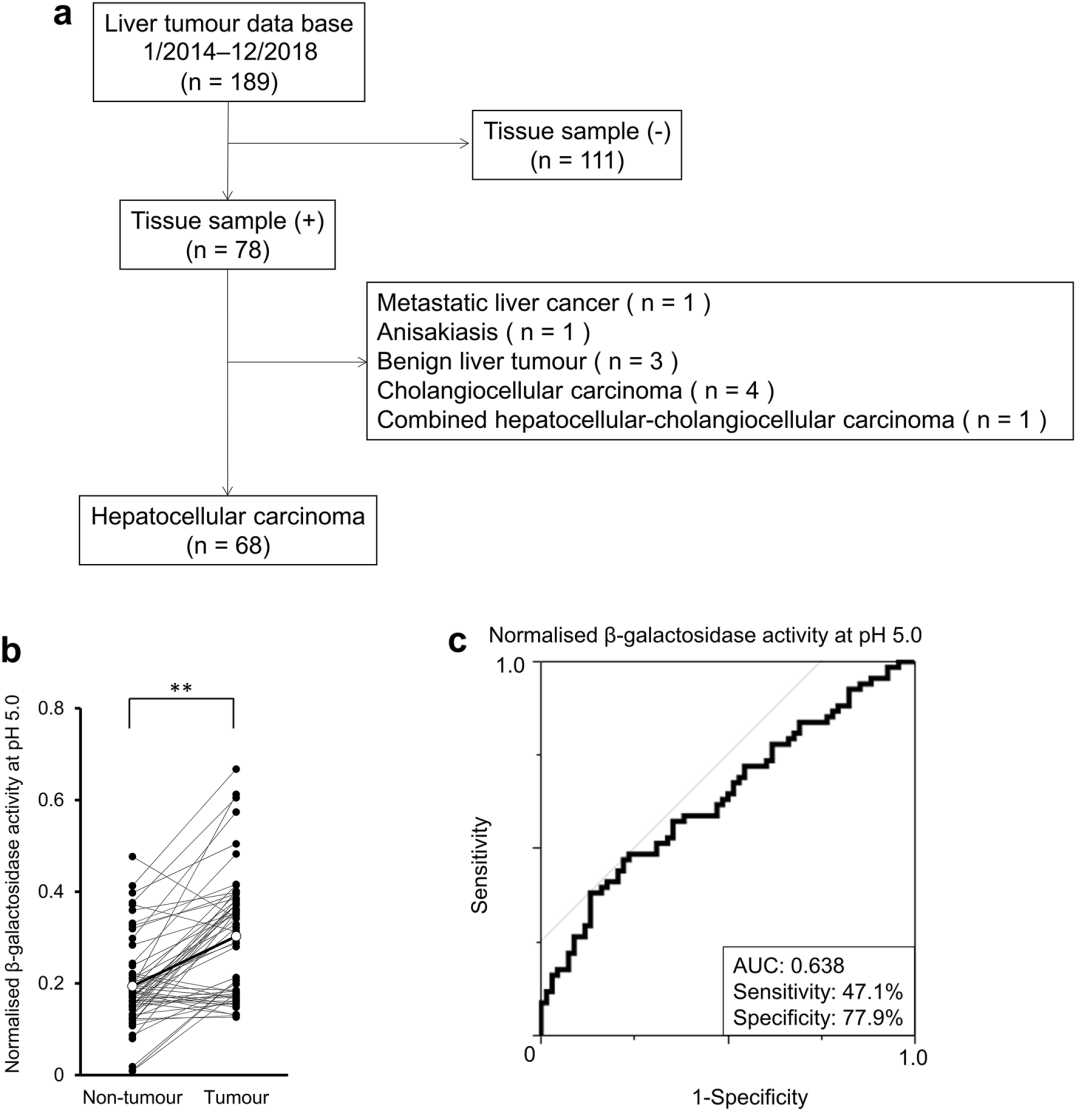
β-galactosidase (β-Gal) activity at pH 5.0 in cryopreserved human hepatocellular carcinoma (HCC) tissue samples. (**a**) Schematic representation of the protocol for the selection of cryopreserved human HCC tissue samples. (**b**) Normalised β-Gal activity at pH 5.0 in tumour and non-tumour liver parenchyma tissues from 68 cryopreserved human HCC samples is shown. Open circle symbols indicate the mean of normalised β-Gal activities in tumour and non-tumour tissues. Normalised β-Gal activity in tumour tissue was significantly higher than that in non-tumour tissue (**p < 0.01). A two-tailed paired *t*-test was used. (**c**) Receiver operating characteristic (ROC) curve of β-Gal activities showing the diagnostic value of measuring β-Gal activity. (AUC: 0.638, Sensitivity: 47.1%, Specificity: 77.9%). The yellow line is a 45° straight line tangent to the ROC curve.

In the present liver tumour database, 68 consecutive patients undergoing liver resection for HCC from January 2014 to December 2018 were selected for this study (Fig. 1a). We examined β-Gal activity at pH 5.0 and pH 7.4 in tumour and non-tumour liver parenchyma from cryopreserved tissue samples from these 68 patients. β-Gal activity at both pH 5.0 (Fig. 1b) and pH 7.4 (Supplementary Fig. S1a) was significantly higher in tumour tissues than that in non-tumour tissues. However, the activity at pH 7.4 was lower than that at pH 5.0 in both tumour and non-tumour tissues. Furthermore, receiver operating characteristics (ROC) curves were generated to determine the sensitivity, specificity, and AUC for β-Gal activity in tumour and non-tumour tissues. At pH 5.0, the sensitivity, specificity, and AUC were 47.1%, 77.9%, and 0.638, respectively (Fig. 1c); at pH 7.4, these values were 40.3%, 88.1%, and 0.630, respectively (Supplementary Fig. S1b). The AUC was almost the same at both pH 5.0 and pH 7.4. Furthermore, we analysed the activity of GGT, a target enzyme of gGlu-HMRG in HCC^22^, and found that GGT activity was significantly lower in tumour tissues than in non-tumour tissues (Supplementary Fig. S2). Based on these results, we performed fluorescence imaging using SPiDER-βGal.

### Association between clinicopathological factors and β-Gal activity

High β-Gal activity in tumour and low β-Gal activity in non-tumour tissues is a prerequisite for cancer-specific fluorescence imaging. Therefore, we determined the clinicopathological factors that could increase β-Gal activity in tumour tissues. β-Gal activity in the tumour tissue of individuals without hepatitis B virus (HBV) and hepatitis C virus (HCV) infections was significantly higher than that in individuals with HBV and HCV infections (Table 1). However, β-Gal activity in non-tumour tissues was not significantly different when assessed based on clinicopathological factors (Table 2). Consequently, β-Gal activity was significantly higher in HCC tissues than in non-tumour tissues; however, no significant association was observed among the clinicopathological factors and β-Gal activity, except for the clinicopathological factors of HBV and HCV infections.

**Table 1:** Association between clinicopathological factors and β-galactosidase (β-Gal) activity in hepatocellular carcinoma (HCC) tumour tissues.

Variable		n	β-galactosidase of tumour median (range)	p-Value
Sex	Male	58	0.214 (0.070–0.667)	0.534
	Female	10	0.262 (0.126–0.612)	
Age	< 70	32	0.285(0.0702–0.67)	0.194
	≥ 70	36	0.208 (0.102–0.612)	
BMI	< 22	24	0.198 (0.070–0.612)	0.266
	≥ 22	44	0.284 (0.102–0.667)	
Maximum tumor size	< 25 mm	34	0.207 (0.070–0.416)	0.261
	≥ 25 mm	34	0.317 (0.103–0.667)	
Differentitation	Well differentiated	24	0.214 (0.102–0.667)	0.532
	Moderately, poorly differentiated	44	0.249 (0.070–0.612)	
Background liver	NL, LC	26	0.202 (0.070–0.574)	0.294
	CH/LF	42	0.313 (0.102–0.667)	
Hepatitis virus	HBV, HCV	27	0.206 (0.070–0.612)	0.049
	Non-B, Non-C	41	0.321 (0.103–0.667)	
AFP	< 10 ng/mL	45	0.199 (0.070–0.605)	0.239
	≥ 10 ng/mL	19	0.313 (0.103–0.667)	
PIVKAII	< 40 mAU/mL	32	0.207 (0.070–0.667)	0.360
	≥ 40 mAU/mL	32	0.309 (0.123–0.612)	
ICG	< 15%	37	0.213 (0.070–0.667)	0.526
	≥ 15%	29	0.215 (0.102–0.504)	
Liver damage	A	65	0.213 (0.070–0.667)	0.550
	B, C	3	0.280 (0.185–0.371)	

β-Gal activity in HCC tumour tissues was analysed in relation to clinicopathological factors. β-Gal activity in the tumour tissue of patients not infected with hepatitis B virus (HBV) and hepatitis C virus (HCV) was significantly higher than that in the tumour tissue of patients infected with HBV and HCV. On analysing other clinicopathological factors (sex, age, BMI, background liver, AFP, PIVKAII, ICG, and grade of liver damage), β-Gal activity in the tumour tissues was not significantly different. A two-tailed Mann–Whitney *U*-test was used to analyse data. BMI; body mass index, NL; normal liver, CH; chronic hepatitis, LF; liver fibrosis, LC; liver cirrhosis, Non-B; patients not infected with HBV, Non-C; patients not infected with HCV, AFP; alpha-fetoprotein, PIVKAII; protein induced by vitamin K absence or antagonist-II, ICG; indocyanine green. Liver damage was defined based on the General Rules for the Clinical and Pathological Study of Primary Liver Cancer, Edition 6, Revised Version^35^.

**Table 2:** Association between clinicopathological factors and β-galactosidase (β-Gal) activity in non-tumour liver parenchyma tissues.

Variable		n	β-galactosidase of non-tumour median (range)	p-Value
Sex	Male	58	0.181 (0.009–0.477)	
	Female	10	0.177 (0.009–0.385)	0.550
Age	< 70	32	0.185 (0.010–0.477)	
	≥ 70	36	0.179 (0.018–0.386)	0.544
BMI	< 22	24	0.175 (0.009–0.385)	
	≥ 22	44	0.184 (0.010–0.477)	0.510
Background liver	NL, LC	26	0.169 (0.009–0.385)	
	CH/LF	42	0.185 (0.010–0.477)	0.284
Hepatitis virus	HBV, HCV	27	0.170 (0.009–0.372)	
	Non-B, Non-C	41	0.187 (0.087–0.477)	0.209
AFP	< 10 ng/mL	45	0.182 (0.009–0.477)	
	≥ 10 ng/mL	19	0.176 (0.010–0.413)	0.537
PIVKAII	< 40 mAU/mL	32	0.175 (0.010–0.413)	
	≥ 40 mAU/mL	32	0.185 (0.009–0.477)	0.529
ICG	< 15%	37	0.187 (0.010–0.413)	
	≥ 15%	29	0.169 (0.009–0.477)	0.381
Liver damage	A	65	0.182 (0.009–0.477)	
	B, C	3	0.172 (0.168–0.194)	0.584

β-Gal activity in non-tumour liver parenchyma tissues was analysed in relation to clinicopathological factors. β-Gal activity in non-tumour was not significantly different when divided into two groups according to clinicopathological factors (sex, age, BMI, background liver, AFP, PIVKAII, ICG, and grade of liver damage). Data were analysed using a two-tailed Mann–Whitney *U*-test. BMI; body mass index, NL; normal liver, CH; chronic hepatitis, LF; liver fibrosis, LC; liver cirrhosis, Non-B; patients not infected with HBV, Non-C; patients not infected with HCV, AFP; alpha-fetoprotein, PIVKAII; protein induced by vitamin K absence or antagonist-II, ICG; indocyanine green. Liver damage was defined based on the General Rules for the Clinical and Pathological Study of Primary Liver Cancer, Edition 6, Revised Version^35^.

### Live cell imaging of cancer cells

**Figure 2:**
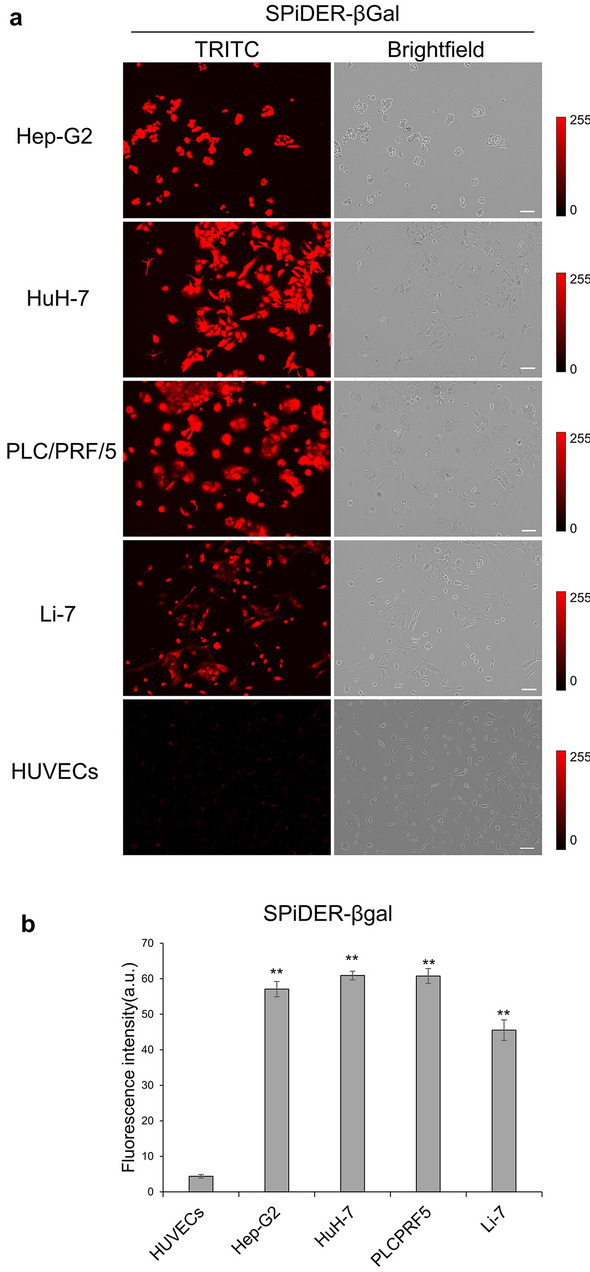
Fluorescence imaging of live cells. (**a**) Fluorescence live cell imaging of hepatocellular carcinoma (HCC) cell lines (Hep-G2, HuH-7, PLC/PRF/5, and Li-7) and HUVECs using SPiDER-βGal (1 μM). Fluorescence was determined using a TRITC filter (left) and Brightfield images (right); images were captured using a Keyence BZ-X800 with a TRITC filter (Excitation: 545/25 nm, Emission: 605/70 nm, Exposure time: 2 s). Scale bar = 100 μm. (**b**) The average fluorescence intensity of 10 randomly selected live cells imaged with SPiDER-βGal was determined using ImageJ (n = 10). Fluorescence intensity was significantly higher in HCC cell lines than in HUVECs (**p < 0.01). A two-tailed Mann–Whitney *U*-test was used. Error bars represent standard error (SE) values.

To investigate the use of SPiDER-βGal, we treated HCC cell lines (Hep G2, HuH-7, PLC/PRF/5, and Li-7) and HUVECs with SPiDER-βGal (1 μM). The intracellular fluorescence signal in HCC cell lines increased after this treatment. However, intracellular fluorescence was low in HUVECs after 60 min of SPiDER-βGal treatment (Fig. 2a). In control HCC cell lines, intracellular fluorescence was almost negligible (Supplementary Fig. S3). Quantification of the fluorescence intensity of 10 randomly selected cells after SPiDER-βGal treatment revealed that the fluorescence intensity of HCC cell lines was significantly higher than that of HUVECs (Fig. 2b).

### Images of tumours in four tumour-bearing mouse models

**Figure 3:**
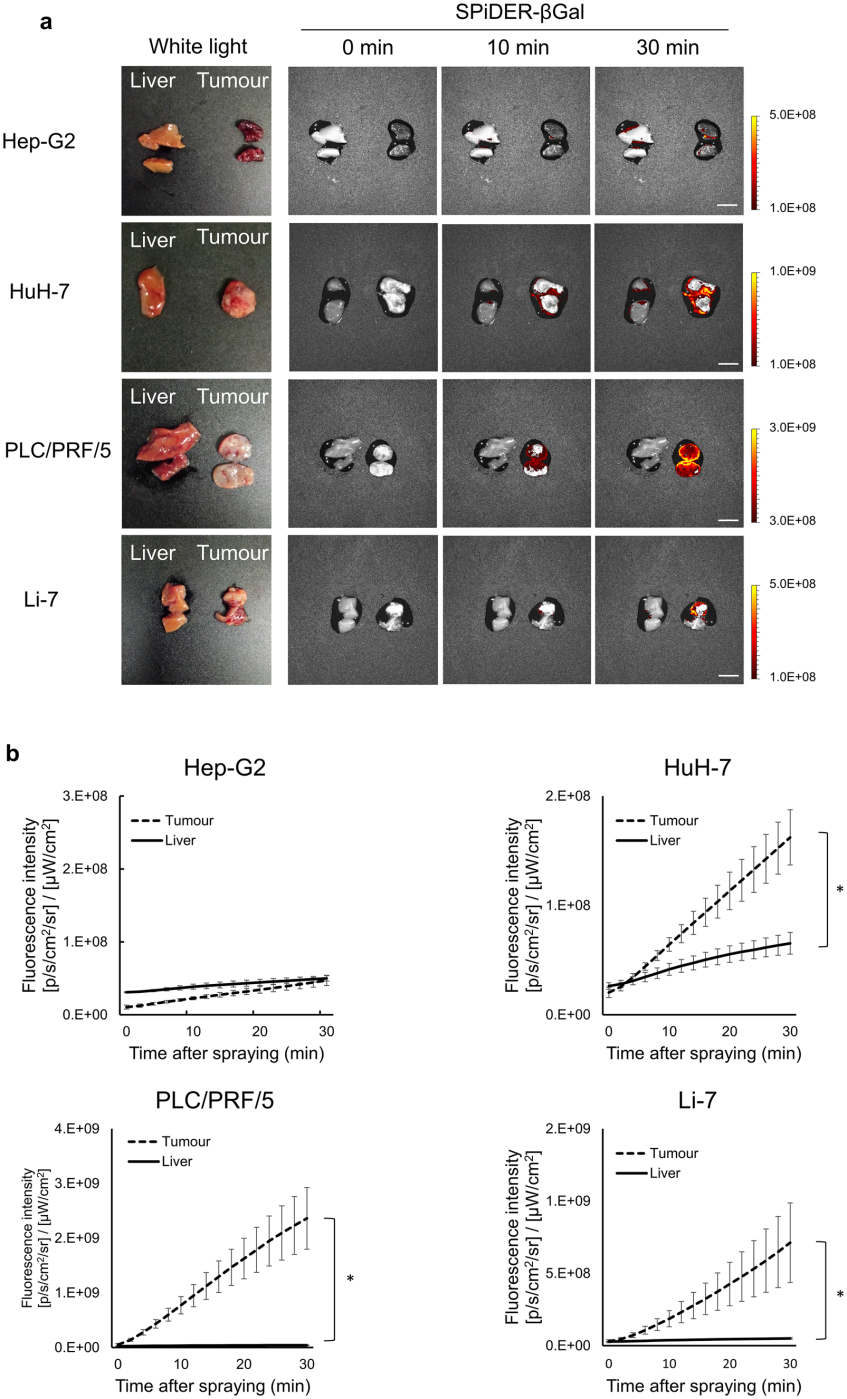
Fluorescence imaging of tumours in four tumour-bearing mouse models. (**a**) Fluorescence images of tumours and normal livers were captured 0, 10, and 30 min after spraying SPiDER-βGal (50 μM) using IVIS Lumina Series III (Excitation: 520 nm, Emission: 570 nm). White light images are also shown. Left: normal liver, Right: tumour. Scale bar = 10 mm. (**b**) Average fluorescence intensity of tumours and normal livers resected from tumour-bearing mice (Hep-G2, n = 3; HuH-7, n = 4; PLC/PRF/5, n = 4; Li-7, n = 4). After 30 min of spraying, HuH-7 tumours, PLC/PRF/5 tumours, and Li-7 tumours had a significantly higher fluorescence intensity than normal liver tissues (*p < 0.05). A two-tailed Mann–Whitney *U*-test was used. Error bars represent standard error (SE) values.

Furthermore, we assessed the potential application of SPiDER-βGal for fluorescence imaging of tumours in four different tumour-bearing mouse models. We successfully established these four tumour mouse models using Hep-G2, HuH-7, PLC/PRF5, and Li-7 cells. After spraying the cut surface of tumours with 50 μM SPiDER-βGal, fluorescent signals were compared with those in normal livers from Hep-G2, HuH-7, PLC/PRF5, and Li-7 tumour-bearing mice (Fig. 3a). In HuH-7, Li-7, and PLC/PRF5 tumour-bearing mice, fluorescence intensity was significantly higher in the tumour 30 min after spraying than that in the normal liver (Fig. 3b). However, in the Hep-G2 tumour-bearing mice, an increase in the fluorescence intensity was almost unobservable in subcutaneous tumours (Fig. 3b). All tumours were confirmed to be cancerous using haematoxylin and eosin staining (Supplementary Fig. S4). Only the subcutaneous tumours in Hep-G2 mouse models indicated a darker red colour when observed with naked eyes (Fig. 3a), and they possessed more red blood cells (RBCs) than the subcutaneous tumours in HuH-7, Li-7, and PLC/PRF5 mouse models in the histopathological analysis (Supplementary Fig. S4).

### Fluorescence imaging of freshly resected human HCC specimens

**Figure 4:**
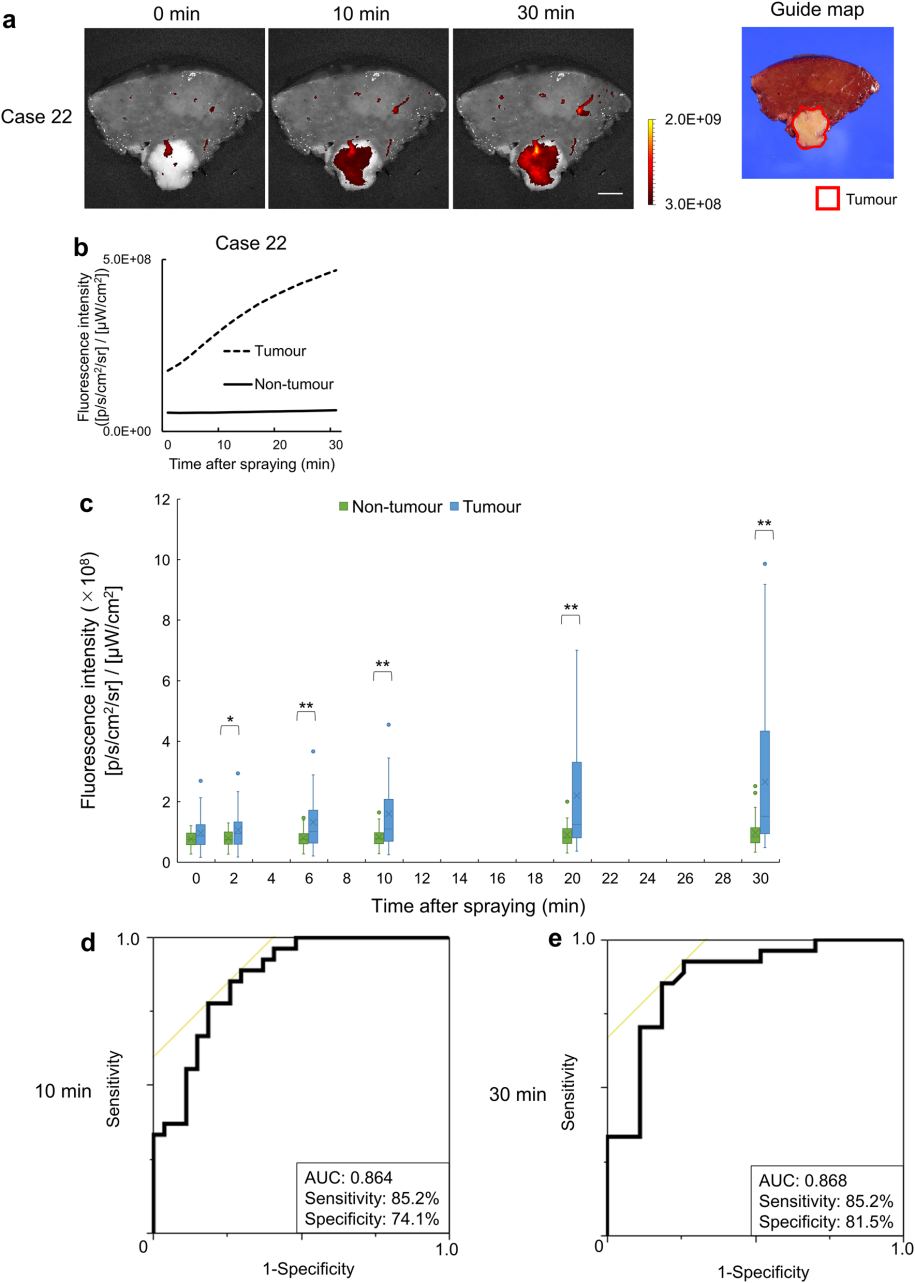
Fluorescence imaging and diagnostic potential of freshly resected human hepatocellular carcinoma (HCC) specimens. (**a**) Representative fluorescence images (case 22) obtained after spraying the specimens with 50 μM SPiDER-βGal, captured using IVIS Lumina Series III (Excitation: 520 nm, Emission: 570 nm). Scale bar = 10 mm. Guide map (white light): The areas surrounded by the red lines indicate the tumour (right). (**b**) Time-dependent changes in fluorescence intensities of tumour and non-tumour tissue after spraying SPiDER-βGal (50 μM) (case 22). (**c**) Box-and-whisker plot of time-dependent changes in fluorescence intensities of the tumour (blue) and non-tumour (green) tissues after spraying 50 μM SPiDER-βGal (HCC, n = 27). Means (cross), medians (horizontal line within box), inter-quartile ranges (box), and ranges (error bars) are indicated. The median fluorescence intensity of the tumour tissues was significantly higher than that of non-tumour tissues after 2 min of spraying SPiDER-βGal (50 μM). *p < 0.05, **p < 0.01. A two-tailed Wilcoxon *t*-test was used. (**d**) Receiver operating characteristic (ROC) curves of the increase in fluorescence intensities from 0 to 10 min. The yellow line is a 45° straight line tangent to the ROC curve. (**e**) ROC curves of the increase in fluorescence intensities from 0 to 30 min. The yellow line is a 45° straight line tangent to the ROC curve.

Freshly resected human HCC samples, obtained from 27 consecutive patients from May 2019 to March 2020, were sprayed with SPiDER-βGal (50 μM) and imaged every 2 min up to 30 min. The patient clinicopathological characteristics are indicated in Supplementary Table S1. Fluorescence intensities were measured in both tumour regions and non-tumour liver regions (Supplementary Fig. S5). Representative fluorescence images (Case 22) are displayed (Fig. 4a). The fluorescence intensity rapidly increased in the tumour, but not in the non-tumour tissue (Fig. 4b). The median fluorescence intensities of tumours in 27 patients were significantly higher than those of non-tumour tissues 2 min after spraying with SPiDER-βGal (50 μM) (Fig. 4c). In tumour samples of 27 patients, the fluorescence intensity increased with time, whereas that of non-tumour tissue displayed limited variation. ROC curves were constructed to determine the diagnostic value of the increase in fluorescence intensity in tumour and non-tumour tissues at 10 min and 30 min. The sensitivity, specificity, and AUC, 10 min after spraying were 85.2%, 74.1%, and 0.864 (Fig. 4d, Supplementary Fig. S6a) and those 30 min after spraying were 85.2%, 81.5%, and 0.868, respectively (Fig. 4e, Supplementary Fig. S6b). A rapid and sufficient diagnostic potential was achieved after 10 min of topically spraying SPiDER-βGal.

## Discussion

Fluorescence-guided surgery is potentially safer and more reliable for liver surgery than conventional surgery. Several previous studies have reported the use of ICG^6–11^ and 5-ALA^13–15^ as fluorescence imaging agents in liver cancers. However, both ICG and 5-ALA requires preoperative administration for cancer imaging. On the other hand, activatable fluorescence probes do not require preoperative preparation and can detect cancers simply through topical spraying, whenever surgeons need to identify cancer. Our current study suggested that SPiDER-βGal is useful for cancer diagnosis in freshly resected human HCC specimens.

In live-cell imaging using SPiDER-βGal, Hep-G2 cells exhibited fluorescence; however, the Hep-G2-derived subcutaneous tumours in mice, which possessed more RBCs than the other cell line-derived subcutaneous tumours, showed a slight increase in fluorescence intensity after application of SPiDER-βGal. Furthermore, haemoglobin reportedly quenches fluorescence^29^. Therefore, haemoglobin in the RBCs of Hep-G2-derived tumours may have influenced the difference observed in the live-cell imaging and mouse model imaging results.

Fluorescence imaging of freshly resected human HCC specimens with SPiDER-βGal is useful; however, the difference in the increase in fluorescence intensity between the tumour and normal liver in a freshly resected human sample during imaging using SPiDER-βGal was greater than the difference in the degree of β-Gal activity of the lysate between the tumour and normal liver. Tumours that showed a slight increase in fluorescence intensity exhibited a reddish colour in white light image (particularly in cases 7, 9, 18, 20, and 25; Supplementary Fig. S5). We also analysed the association between other clinicopathological factors and the increase in fluorescence intensity within the tumour (Supplementary Table S2). There were no statistical differences between each clinicopathological factor. Thus, the colouration of tissue samples may influence the fluorescence intensity, as in our mouse model imaging of the Hep-G2-derived tumour.

In a previous study, gGlu-HMRG, a GGT-targeted fluorescence probe, has been reported as a useful tool for the detection of liver cancers^22^. We also measured the activity of GGT in cryopreserved human HCC and liver parenchyma tissue samples and found that it was not upregulated in tumour tissues, compared with that in non-tumour tissues (Supplementary Fig. S2). Based on the results, it was suggested that the colouration of tissue samples may also have influenced the results of fluorescence imaging with gGlu-HMRG, in line with the observations for SPiDER-βGal.

In our fluorescence imaging analysis of freshly resected human HCC specimens using SPiDER-βGal, fluorescence was observed not only in tumour tissues but also in the vasculature, such as in Glisson’s capsule or hepatic veins (particularly in cases 3, 4, 7, 14, and 27; Supplementary Fig. S5). Although the β-Gal activity of these vasculatures was not evaluated, we need to be careful when analysing the clinical images because of the potential of the fluorescent vessels that may result in false-positive results.

β-Gal is present in the lysosome and is active in an acidic environment. In resected specimens, blood flow is disrupted; hence, it is difficult to determine if the cellular and organellar pH is maintained. We analysed β-Gal activity in cryopreserved human HCC tissues at pH 7.4 and 5.0 and found that β-Gal activity was lower at pH 7.4 than at pH 5.0 in both tumour and non-tumour tissues. At pH 7.4, the enzyme activity was significantly higher in tumour tissues than that in non-tumour tissues. The AUC of β-Gal activity at pH 7.4 was almost the same as that at pH 5.0. Based on differences between tumour and non-tumour tissues, we determined that changes in pH had limited effects on the outcomes of fluorescence imaging.

Tumour β-Gal activity has been reported to be especially high in breast cancer, colon cancer^30^, and gliomas^31^. With respect to the β-Gal activity in HCC, one study using 5-bromo-4-chloro-3-indolyl β-D-galactopyranoside (X-Gal) staining has reported that some HCC liver tumour cells express β-Gal^32^; however, details of β-Gal activity in HCC remains unknown. Our results suggested that individuals without HBV and HCV infections have high β-Gal activity in tumour tissues. HCV core protein was reported to inhibit HCC cell replicative senescence^33^. Further, β-Gal is known to be a senescence marker^34^. So, β-Gal activity may be influenced by a hepatitis virus infection. In order to improve the diagnostic performance of the β-Gal-targeted fluorescence probe, further study of the association between the hepatitis virus and β-Gal are necessary in the future.

Thus, this study indicates that SPiDER-βGal is useful for the rapid fluorescence imaging of human HCC. SPiDER-βGal might be applicable for intraoperative diagnosis. In particular, fluorescence-guided surgery using SPiDER-βGal is considered useful for determining whether the tumour is exposed at the resected liver surface. Fluorescence-guided surgery with SPiDER-βGal might facilitate complete liver resection and reduce HCC recurrence.

## Methods

### Cryopreserved human HCC tissue samples

Tissue samples from the site of tumour and non-tumour liver parenchyma were harvested and cryopreserved from patients undergoing a curative liver resection at the University Hospital of Kyoto Prefectural University of Medicine (KPUM). A consort flow chart is provided in Fig. 1a. The liver tumour database searched from January 2014 to December 2018 revealed 189 patients. The 111 patients, from whom tissue samples could not be harvested, were excluded. Of the remaining 78 patients, those with metastatic liver cancer (n = 1), anisakiasis (n = 1), benign liver tumour (n = 3), cholangiocellular carcinoma (n = 4), and combined hepatocellular-cholangiocellular carcinoma (n = 1) were excluded. Finally, 68 consecutive patients were included in this study. The Institutional Review Board of KPUM examined and approved the study protocol (approval number: ERB-C-67) in accordance with the tenets of the Declaration of Helsinki. Written informed consent was acquired preoperatively from all patients. Among the 68 patients, alpha-fetoprotein (AFP) and protein induced by vitamin K absence or antagonist-II (PIVKAII) could not be measured in four patients and two patients did not have ICG data.

### Cell lines and culture

Four HCC cell lines and human umbilical vein endothelial cells (HUVECs) were used in this study: Hep-G2, HuH-7, and Li-7 cell lines (RIKEN Bioresource Center, Japan); PLC/PRF/5 cell line (JCRB Cell Bank, Japan); HUVECs (provided by T.K., Tokyo, Japan). HuH-7, Hep-G2, and PLC/PRF/5 cells were cultured in DMEM (Nacalai Tesque, Inc.,Japan). Li-7 cells were cultured in RPMI1640 (Nacalai Tesque) Both medium contained 10% heat-inactivated foetal bovine serum (Gibco, MA, USA), penicillin (100 U/mL) and streptomycin (100 μg/mL) (Nacalai Tesque). HUVECs were cultured in Endothelial Cell Basal Medium-2 (Lonza, Switzerland) and Endothelial Cell Growth Medium-2 SingleQuots^[^™^]^ Supplements and Growth Factors (Lonza) in collagen I-coated dishes (AGC TECHNO GLASS, Japan). All cells were cultured at 37 °C in a humidified atmosphere containing 5% CO_2_.

### Preparation of cryopreserved HCC tissue lysates

Cryopreserved tissues were suspended in CelLytic M (Sigma-Aldrich, MO, USA) and finely chopped using scissors. Thereafter, the chopped tissues were homogenised using an ultrasonic homogeniser on ice. The lysed tissues were centrifuged (14,000 × *g* for 10 min at 4 °C) to pellet cellular debris. The supernatant was then harvested and assessed for protein concentration using a BCA protein assay kit (Pierce, MA, USA), in accordance with the manufacturer’s instructions. Subsequently, the supernatant was diluted to 1 mg/mL with D-PBS (Nacalai Tesque).

### Evaluation of β-Gal activity

β-Gal activity was evaluated in 96-well black plates (CORNING, MA, USA) using acetate buffer (pH 5.0) (Nacalai Tesque) or D-PBS (pH 7.4) and a FluoReporter lacZ/Galactosidase Quantitation Kit (Thermo Fisher Scientific, MA, USA), in accordance with the manufacturer’s instructions. First, tissue lysate samples (1 mg/mL protein concentration, 10 μL/well) were added to triplicate wells. Thereafter, 3-carboxyumbelliferyl β-D-galactopyranoside (1.1 mM) prepared in acetate buffer (pH 5.0; 100 μL/well) or D-PBS (pH 7.4) was added to the wells. Acetate buffer (10 μL/well) and 7-hydroxycoumarin-3-carboxylic acid (0.1 mM) diluted with acetate buffer (100 μL/well) or D-PBS (pH 7.4) (10 μL/well) and 7-hydroxycoumarin-3-carboxylic acid (0.1 mM) diluted with D-PBS (100 μL/well) were added to triplicate wells as a reference standard. For the β-Gal activity assay at pH 5.0, the plates were incubated for 30 min (5% CO_2_, 37 °C). Then, 50 μL of Na_2_CO_3_ (0.2 M in H_2_O) was added to all wells to terminate the reaction and the fluorescence intensity (Ex/Em: 390/460 nm) was measured using a micro plate reader (SpectraMax M2, Molecular Devices, CA, USA). For the β-Gal activity assay at pH 7.4, the plate was incubated for 30 min (5% CO_2_, 37 °C). Subsequently, the fluorescence intensity was measured. The intensity of each sample was normalised against that of the reference standard.

### GGT activity assay

GGT activity was evaluated in 384-well black plates using fluorescence probes (gGlu-HMRG and HMRG). Tissue lysates (1 mg/mL protein concentration, 5 μL/well) and fluorescence probes (1.33 μM in D-PBS, final: 1 μM) were added to each well. HMRG was used as a reference standard, and gGlu-HMRG was used as a fluorescence probe. Fluorescence intensity was measured using an EnVision multilabel plate reader (Perkin Elmer, MA, USA) every minute for 120 min (FITC filter; Ex/Em: 485/535 nm). The results of the gGlu-HMRG assay were normalised to those of the HMRG assay, which was concurrently performed. GGT activity was determined using the following formula:

$${\text{Activity = (fluorescence increase rate) / (fluorescence intensity of HMRG in lysate}} - {\text{ fluorescence intensity of gGlu - HMRG just after lysate addition) / (protein concentration)}}{\text{.}}$$

### Live cell imaging

Cells (1.0 × 10^4^ cells/dish) were seeded in the centre of 35-mm glass-bottom dishes (Matsunami glass, Japan) and incubated in an atmosphere containing 5% CO_2_ at 37 °C for 1–2 d. Thereafter, the cells were washed twice with Hanks’ Balanced Salt solution (HBSS; Nacalai Tesque). Next, SPiDER-βGal (1 μM) was added to the dishes, and the cells were incubated in 5% CO_2_ at 37 °C for 60 min. Fluorescent images were obtained using a Keyence BZ-X800 with the TRITC filter (Ex: 545/25 nm, Em: 605/70 nm, Exposure time: 2 s). Bright-field images were captured simultaneously. As a control, we added an identical volume of HBSS to the dishes of cultured cells instead of SPiDER-βGal. The fluorescence intensities of 10 randomly selected cells were analysed using Image J version 1.52a (NIH).

### Mouse model imaging

All animal experiments were performed in compliance with both the ARRIVE guidelines and the institutional guidelines of KPUM, and approved by the animal experimental committee of KPUM (approval number: M30-554). Five-week-old female BALB/c nu/nu mice (average weight, 17 g) were purchased from SHIMIZU Laboratory Supplies, Japan. The mice were housed in plastic cages with stainless-steel grid tops in an air-conditioned environment with a 12-h light–dark cycle and were fed regular food and water ad *libitum*. Individual suspensions of four types of HCC cells in D-PBS (2.0 × 10^7^ cells/mL) were mixed with an equal amount of Matrigel (CORNING, MA, USA) on ice. Under general anaesthesia, the mixed suspension (100 μL) was then subcutaneously injected into the flanks of each mouse. After ≥ 4 weeks, tumour-bearing mice were euthanised using Isoflurane (Wako, Japan). Subcutaneous tumours and livers were dissected and divided into two using scissors. A solution of SPiDER-βGal (50 μM) in HBSS was then sprayed onto the cut surface of each tumour or the surface of normal liver. Sequence of fluorescent images were captured every 2 min for 30 min using IVIS Lumina Series III (Ex/ Em: 520/570 nm). Regions of interest (ROIs) were drawn for both the tumour and normal liver tissues, and the average radiant efficiency was determined as fluorescence intensity using Living Image version 4.4.

### Histopathological analysis

Resected subcutaneous tumours from tumour-bearing mice were fixed with 10% neutral buffered formalin then embedded in paraffin. The paraffin blocks were sliced to a thickness of 5 μm, after which the paraffin-embedded sections were deparaffinised and stained with Mayer’s haematoxylin solution (Wako) and eosin Y (Wako) for histopathological analysis.

### Freshly resected human specimens

Freshly resected human specimens were obtained from patients preoperatively diagnosed with HCC—through radiological examination—who received curative liver resection at the University Hospital of KPUM. Written informed consent was preoperatively acquired from all patients. The Institutional Review Board of KPUM examined and approved the research procedures (approval number: ERB-C-1470) in accordance with the tenets of the Declaration of Helsinki. Cases of cholangiocellular carcinoma without HCC components, as determined pathologically, were excluded. In total, 27 freshly resected human HCC specimens were examined prospectively from May 2019 to March 2020. Patient clinicopathological characteristics were described based on the General Rules for the Clinical and Pathological Study of Primary Liver Cancer, Edition 6, Revised Version^35^.

### Human specimen imaging

Fluorescence imaging of human specimens was performed within 1 h after liver resection. A solution of SPiDER-βGal (50 μM) prepared in HBSS was then sprayed onto the resected surface of the liver tissue samples. Sequences of fluorescent images were captured every 2 min for 30 min using IVIS Lumina Series III (Ex/Em: 520/570 nm). ROIs were drawn for both the tumour and normal liver, and the average radiant efficiency as a fluorescence intensity was determined using Living Image version 4.4.

### Statistical analysis

A two-tailed paired *t*-test was used to compare β-Gal activity in cryopreserved human HCC samples at pH 5.0 and pH 7.4, and GGT activity in cryopreserved human HCC samples. A two-tailed Mann–Whitney *U*-test was used to compare the fluorescence intensity of live cells, fluorescence intensity of images from tumour-bearing mouse models, and clinicopathological characteristics of human HCC tissue samples that were either cryopreserved or freshly resected. A two-tailed Wilcoxon *t*-test was used to compare the fluorescence intensity of freshly resected HCC specimens. ROC curves were constructed using JMP13 (SAS Institute, NC, USA). ROC curves and Youden index were used to determine the optimal cut-off value for β-Gal activity in the cryopreserved human HCC samples and for assessing the increase in fluorescence intensity in freshly resected HCC specimens. The sensitivity, specificity, and area under the curve (AUC) were determined through ROC analysis. Results with *p*-values < 0.05 were considered significant. Statistical analysis was performed using the yStat 2013 software.

## Supplementary Information

Supplementary Information.

## Data Availability

The datasets of the current study are available from the corresponding author on reasonable request.
