# Supplementary material for: Rapid fluorescence imaging of human hepatocellular carcinoma using the β-galactosidase-activatable fluorescence probe SPiDER-βGal
Source: Sci Rep. 2021 Sep 9;11:17946. doi: 10.1038/s41598-021-97073-1 (PMC8429424; doi:10.1038/s41598-021-97073-1)
Supplement: Supplementary file 1 — Supplementary Information. [file 41598_2021_97073_MOESM1_ESM.docx]

**Supplementary Data**

**Rapid fluorescence imaging of human hepatocellular carcinoma**

**using the β-galactosidase-activatable fluorescence probe SPiDER-βGal**

Soichiro Ogawa^1^, Hidemasa Kubo^1*^, Yasutoshi Murayama^1^, Takeshi Kubota^1^

Masayuki Yubakami^1^, Tatsuya Matsumoto^1^, Yusuke Yamamoto^1^, Ryo Morimura^1^

Hisashi Ikoma^1^, Kazuma Okamoto^1^, Mako Kamiya^2^, Yasuteru Urano^2,3,4^, Eigo Otsuji^1^

^1^Division of Digestive Surgery, Department of Surgery, Kyoto Prefectural University of Medicine, 465 Kajii-cho, Kamigyo-ku, Kyoto, 602-8566, Japan

^2^Graduate School of Pharmaceutical Sciences, The University of Tokyo, 7-3-1 Hongo, Bunkyo-ku, Tokyo 113-0033, Japan

^3^Graduate School of Pharmaceutical Sciences, The University of Tokyo, 7-3-1 Hongo, Bunkyo-ku, Tokyo 113-0033, Japan

^4^CREST (Japan) Agency for Medical Research and Development (AMED), 1-7-1 Otemachi, Chiyoda-ku, Tokyo 100-0004, Japan

***Correspondence to:**

Hidemasa Kubo

Division of Digestive Surgery, Department of Surgery, Kyoto Prefectural University of Medicine, 465 Kajii-cho, Kamigyo-ku, Kyoto, 602-8566, Japan,

Phone: +81-75-251-5527; Fax: +81-75-251-5522; E-mail: h-kubo@koto.kpu-m.ac.jp

**Supplementary Figures**

**
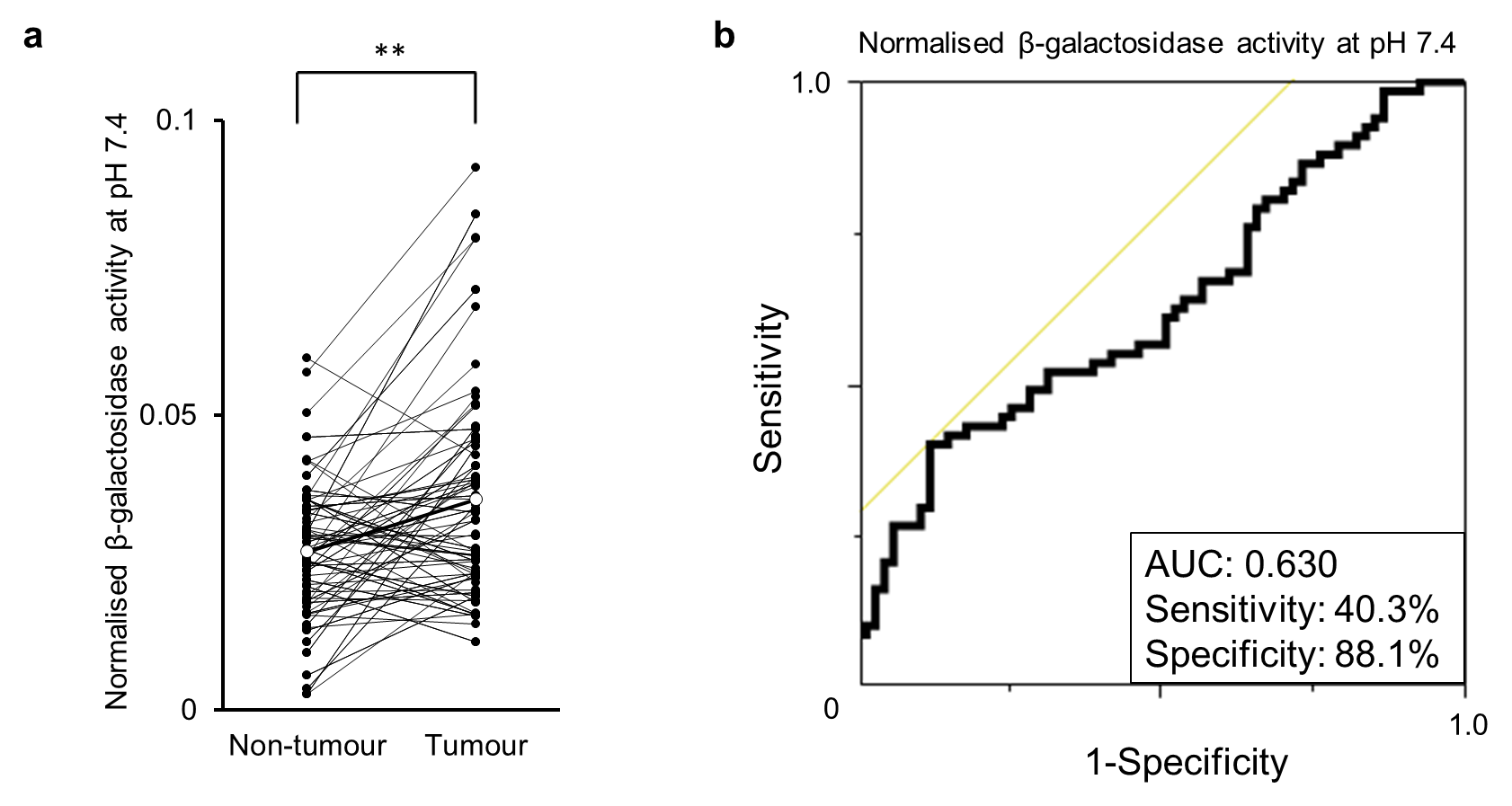
**

**Supplementary Fig. S1**

1. Normalised β-galactosidase (β-Gal) activities evaluated at pH 7.4 in tumour and non-tumour tissues from 67 cryopreserved clinical hepatocellular carcinoma (HCC) tissues. Open circles indicate the mean of normalised β-Gal activity in tumour and non-tumour tissues. Normalised β-Gal activity in tumour tissue was significantly higher than that in non-tumour tissue (***p* < 0.01). A two-tailed paired *t*-test was used.
2. Receiver operating characteristic (ROC) curves of β-Gal activities at pH 7.4 showing the diagnostic value of measuring β-Gal activity. The yellow line is a 45° straight line tangent to the ROC curve. (AUC: 0.630, Sensitivity: 40.3%, Specificity: 88.1%)

**
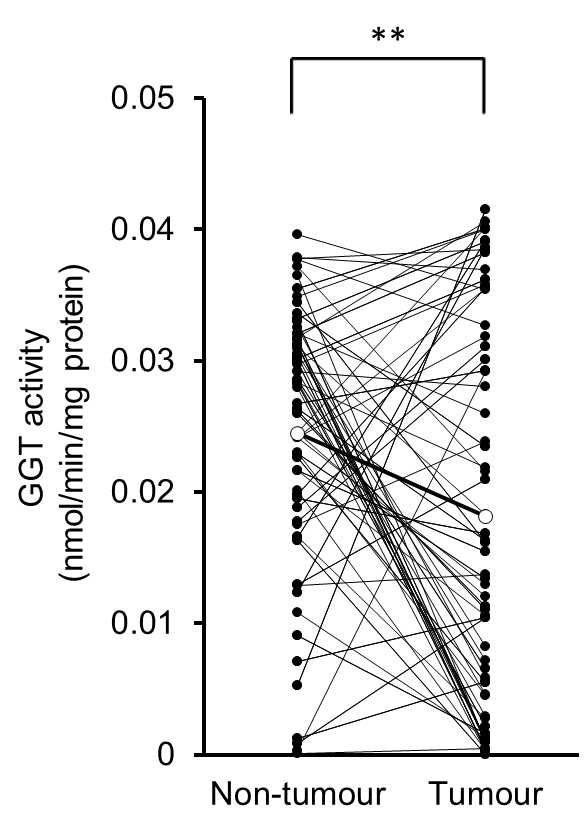
**

**Supplementary Fig. S2**

Gamma-glutamyl transpeptidase (GGT) activity in tumour and non-tumour tissues from 67 cryopreserved clinical hepatocellular carcinoma samples. Open circles indicate the mean GGT activity in tumour and non-tumour tissues. GGT activity in tumour tissues was significantly lower than that in non-tumour tissues (***p* < 0.01). A two-tailed paired *t*-test was used.


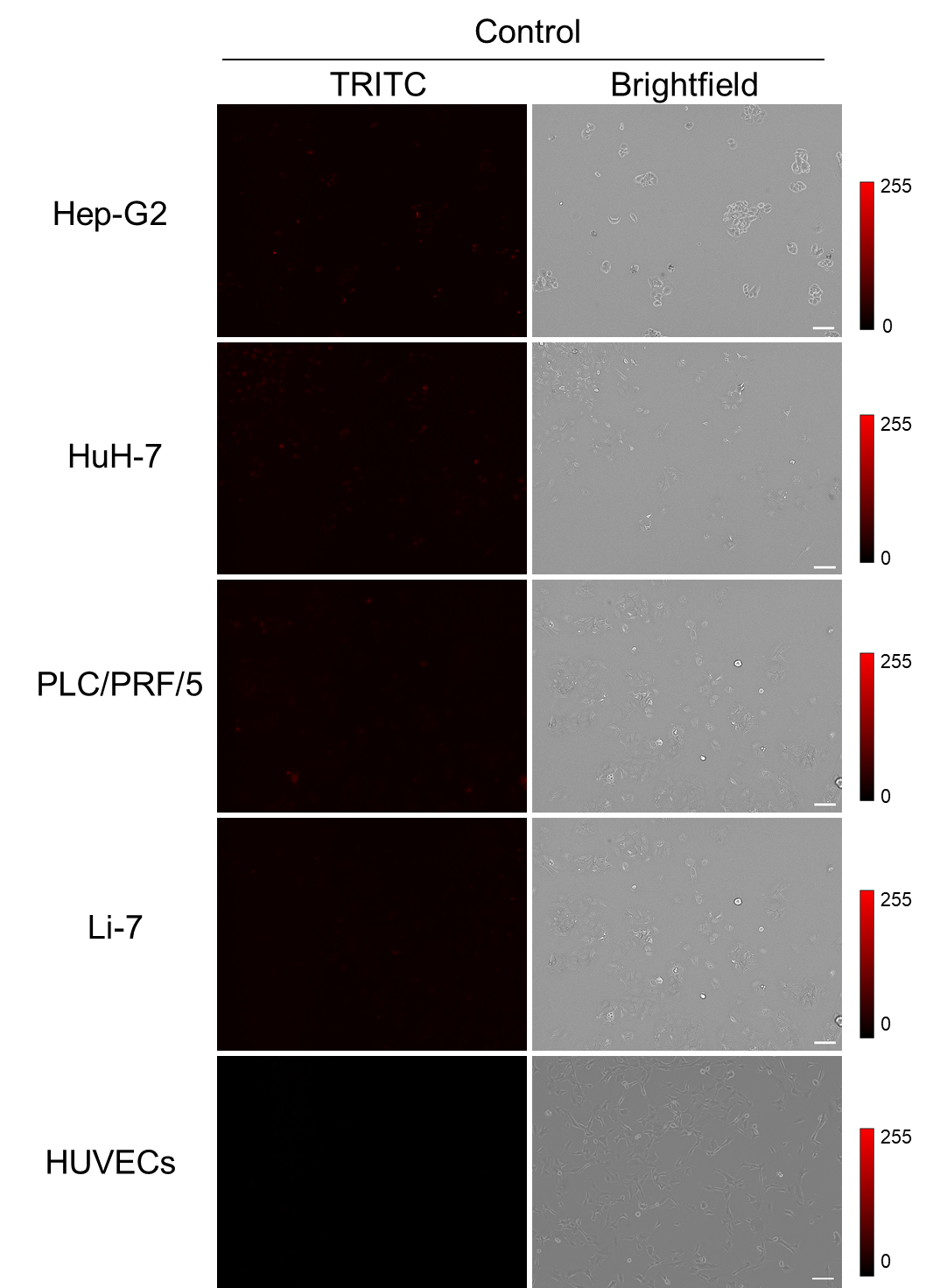


**Supplementary Fig. S3**

Fluorescence live-cell imaging of hepatocellular carcinoma (HCC) cell lines (Hep-G2, HuH-7, PLC/PRF/5, and Li-7) and human umbilical vein endothelial cells (HUVECs) with Hanks’ Balanced Salt solution (HBSS) as a control. Fluorescence was determined using a TRITC filter (left) and Brightfield images (right); images were captured using a Keyence BZ-X800 with a TRITC filter (Excitation: 545/25 nm, Emission: 605/70 nm, Exposure time: 2 s). Scale bar = 100 μm.

**
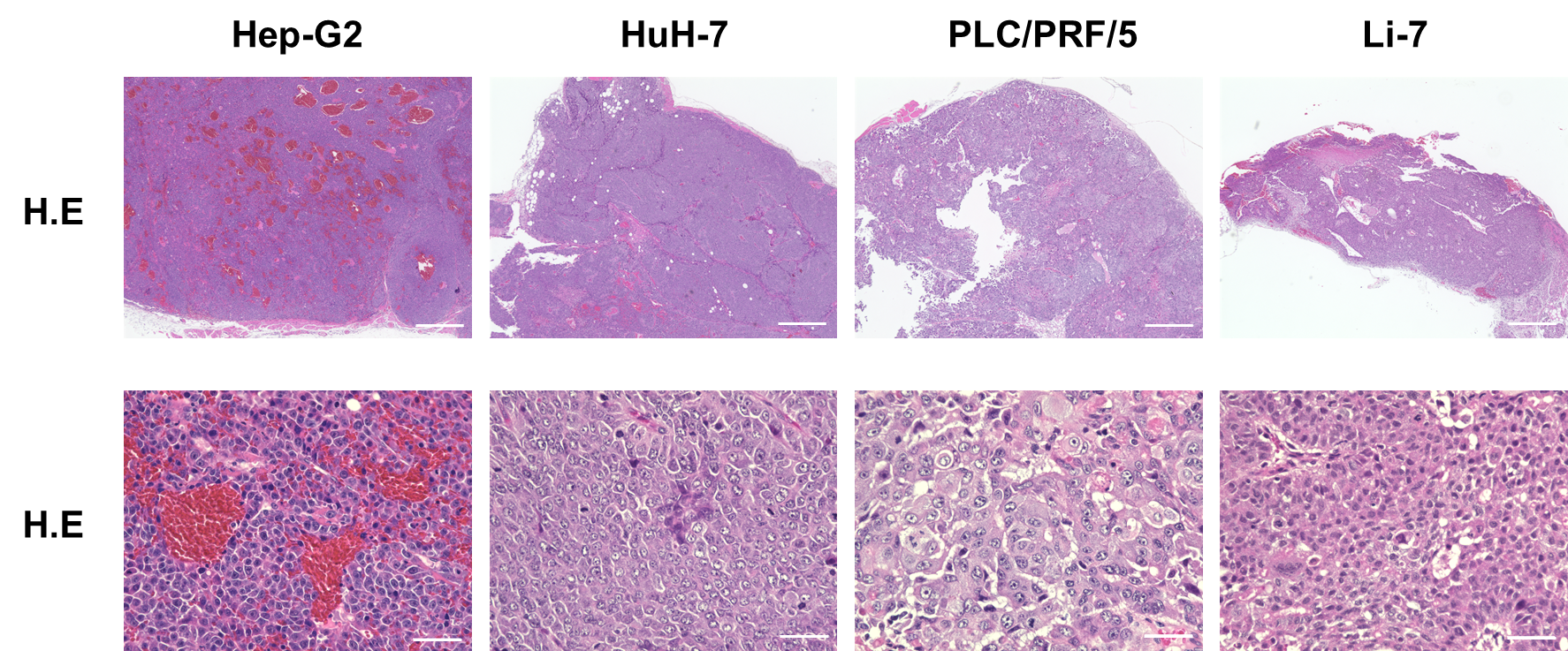
**

**Supplementary Fig. S4**

Histopathological findings of tumours resected from mice injected with four hepatocellular carcinoma cell types (Hep-G2, HuH-7, PLC/PRF/5, and Li-7). Samples were stained with haematoxylin and eosin (H.E). Tumours resected from all tumour-bearing mice were confirmed to be cancerous. Subcutaneous tumours composed of Hep-G2 cells contained more red blood cells than those composed of HuH-7, PLC/PRF/5, and Li-7 cells. Above: scale bar = 500 μm. Bottom: scale bar = 20 μm.

**
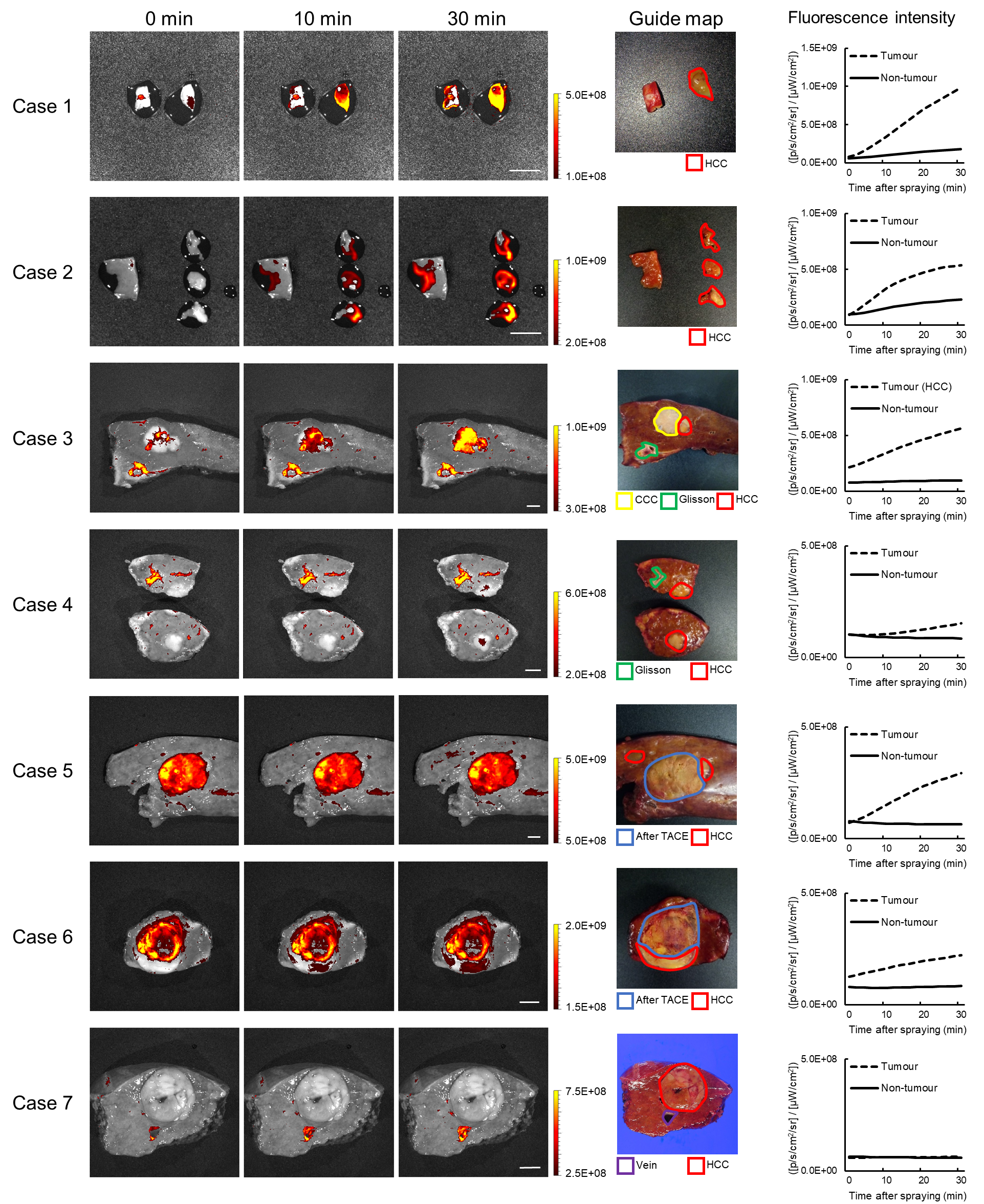
**

**Supplementary Fig. S5a**

Fluorescence images after spraying with SPiDER-βGal at 0, 10, and 30 min, guide map (white light), and time-dependent changes in the fluorescence intensities of tumours and non-tumour tissues with time (Cases 1–7). Areas surrounded by red, blue, yellow, green, and purple lines indicate hepatocellular carcinoma (HCC), after transcatheter arterial chemo-embolisation (TACE), cholangiocellular carcinoma (CCC), Glisson’s capsule (Glisson), and hepatic vein (Vein), respectively. Scale bar = 10 mm.

**
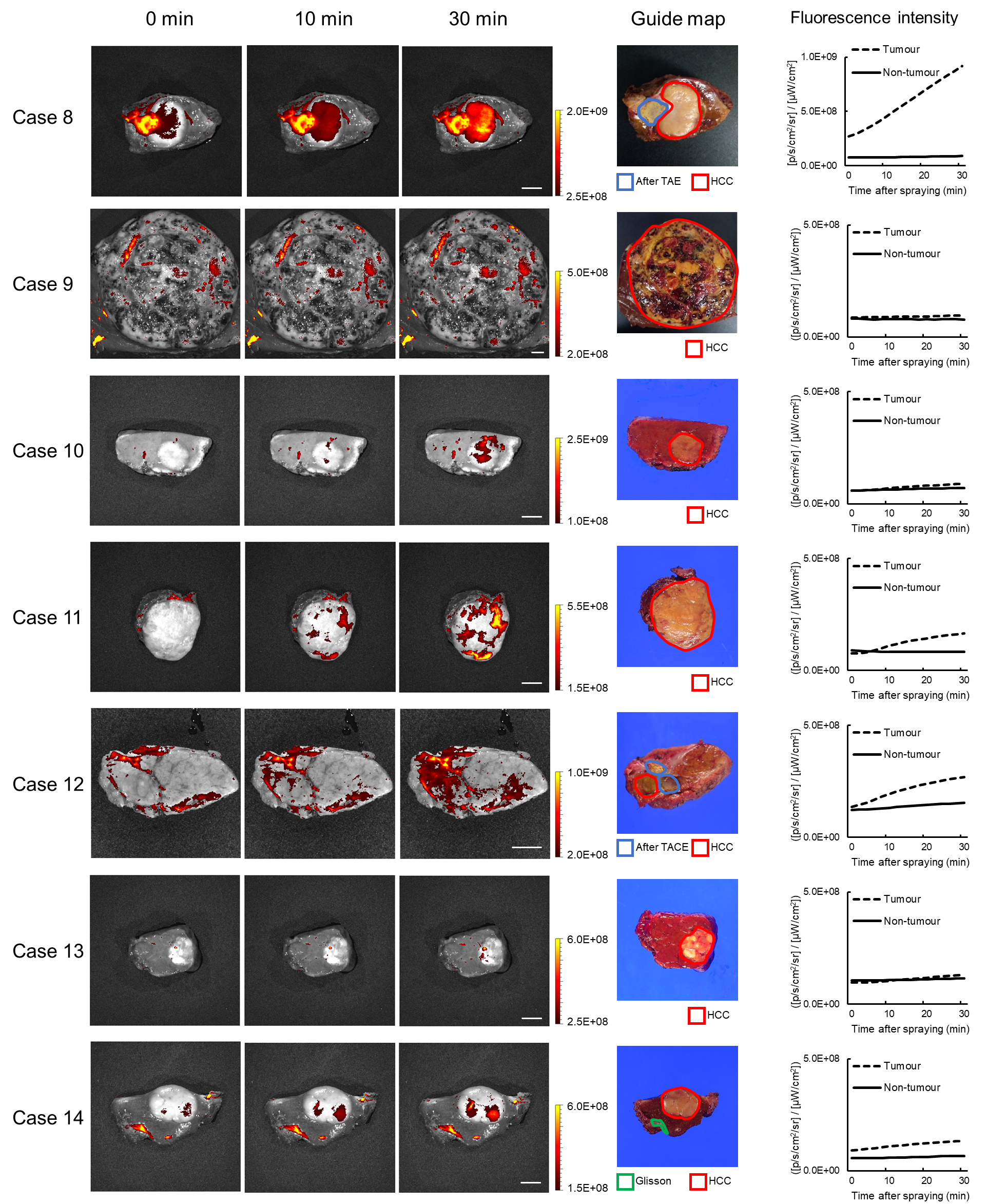
**

**Supplementary Fig. S5b**

Fluorescence images after spraying with SPiDER-βGal at 0, 10, and 30 min, guide map (white light), and time-dependent changes in the fluorescence intensities of tumours and non-tumour tissues with time (Cases 8–14). Areas surrounded by red, blue, and green lines indicate HCC, after transcatheter arterial embolisation (TAE), TACE, and Glisson, respectively. Scale bar = 10 mm.

**
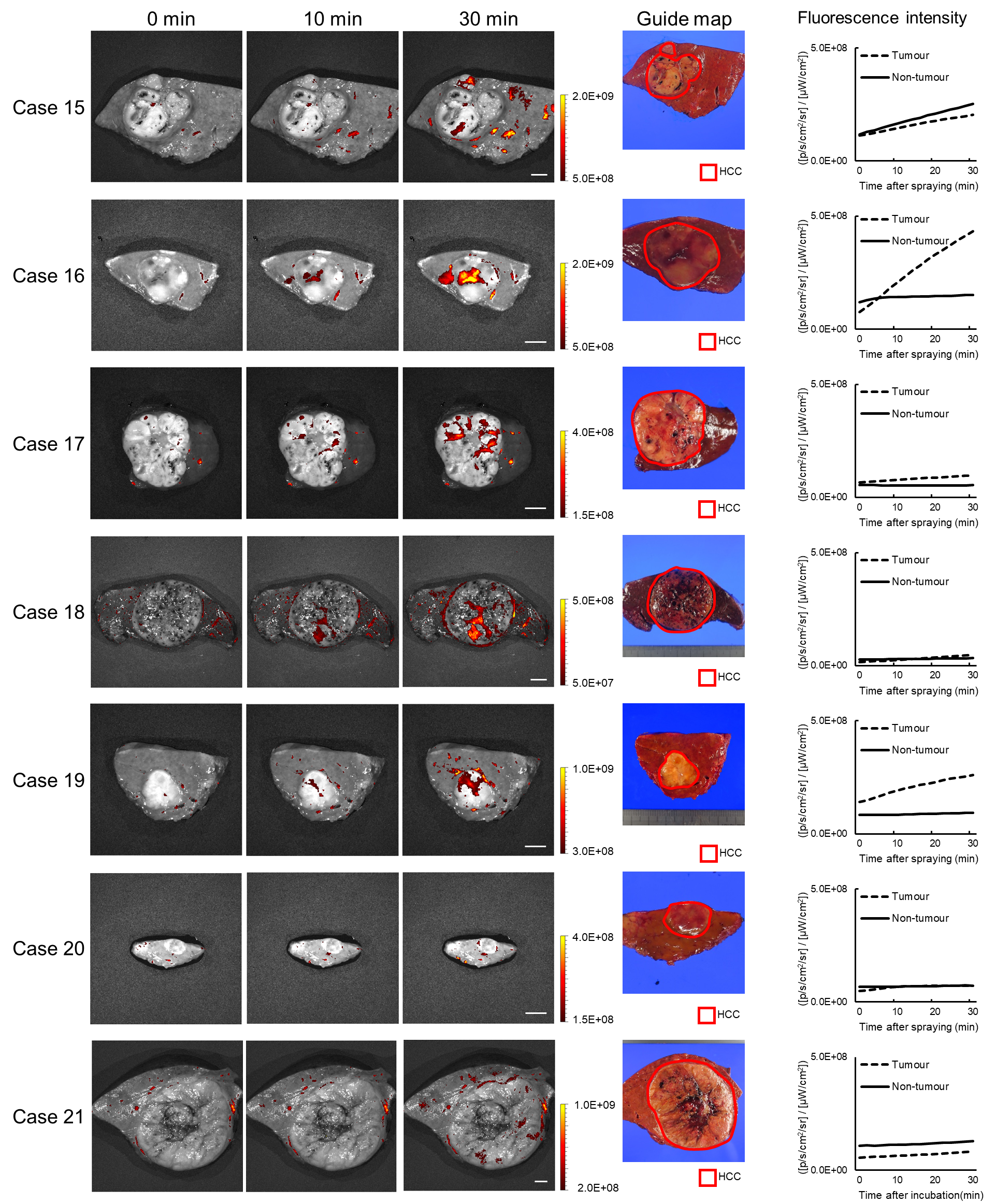
**

**Supplementary Fig. S5c**

Fluorescence images after spraying with SPiDER-βGal at 0, 10, and 30 min, guide map (white light), and time-dependent changes in the fluorescence intensities of tumours and non-tumour tissue with time (Cases 15–21). Areas surrounded by red lines indicate HCC. Scale bar = 10 mm.


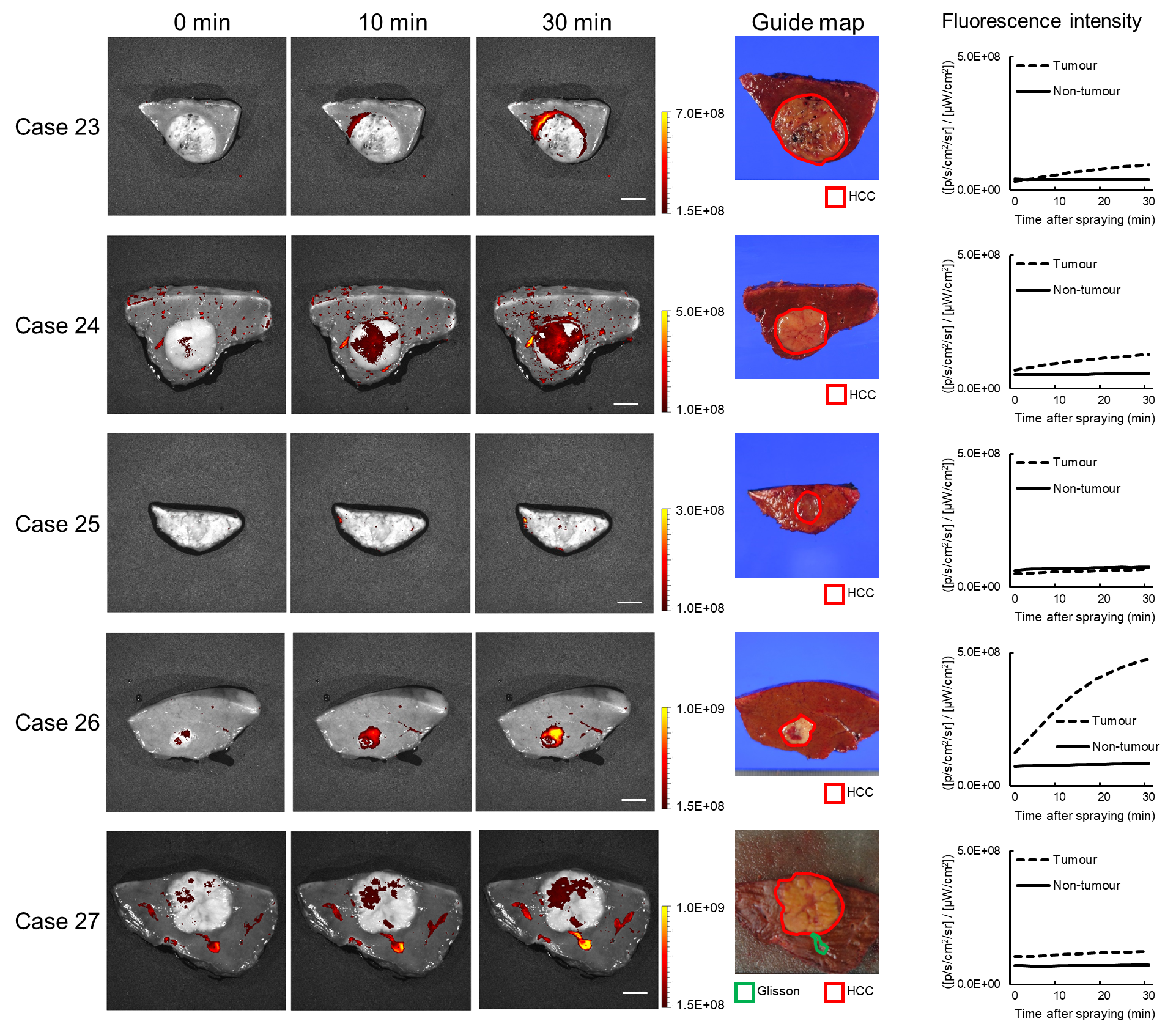


**Supplementary Fig. S5d.**

Fluorescence images after spraying with SPiDER-βGal at 0, 10, and 30 min, guide map (white light), and time-dependent changes in the fluorescence intensities of tumours and non-tumour tissue with time (Cases 23–27). Areas surrounded by red and green lines indicate HCC and Glisson, respectively. Scale bar = 10 mm.


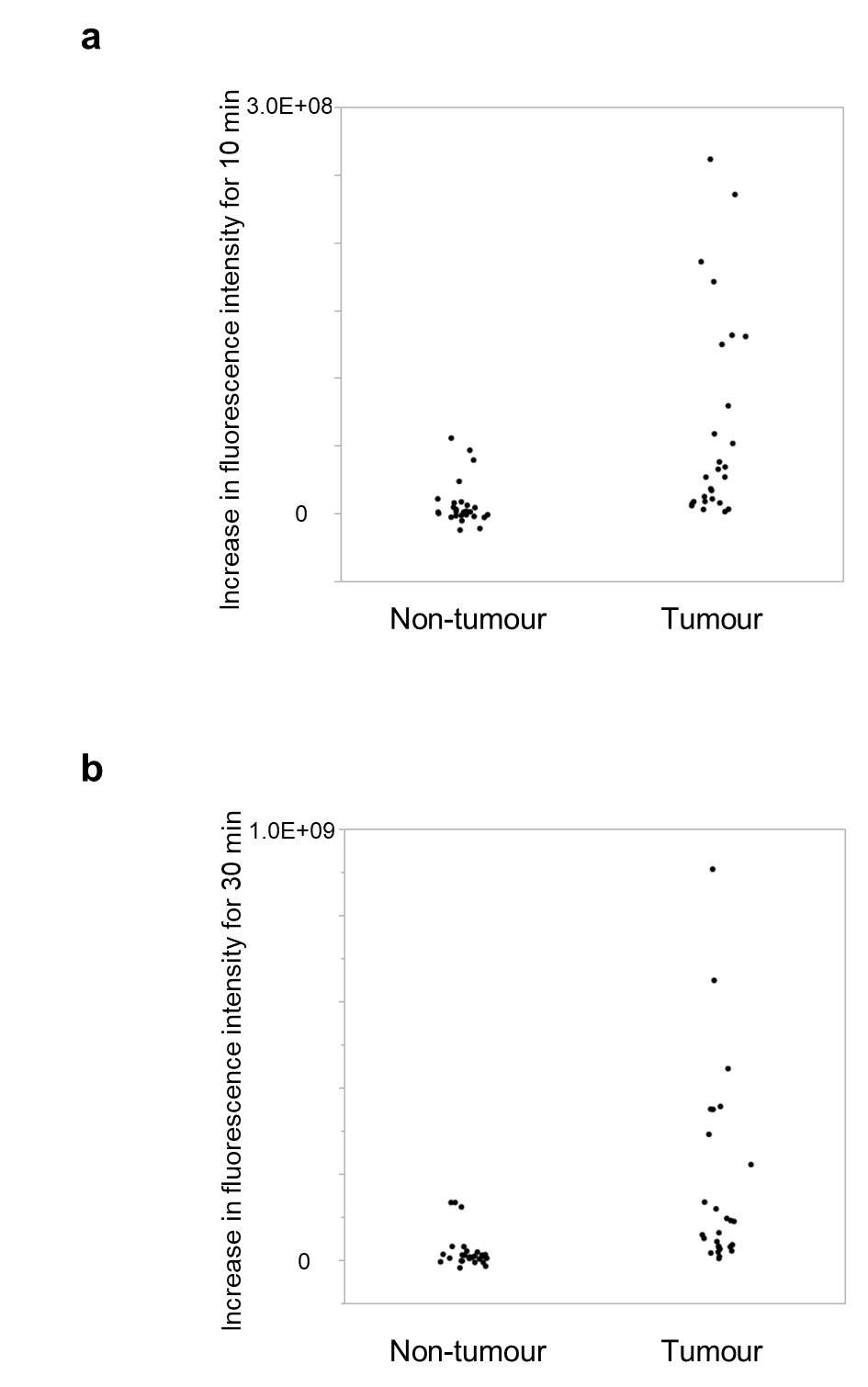


**Supplementary Fig. S6**

Scatter plots of the increase in fluorescence intensities in tumour and non-tumour tissues.

1. From 0 min to 10 min.
2. From 0 min to 30 min.

**Supplementary Tables**

**Supplementary Table S1.** The clinicopathological characteristics of 27 patients described in accordance with The General Rules for the Clinical and Pathological Study of Primary Liver Cancer, Edition 6, Revised Version.

NBNC; non-B, non-C hepatitis, B+; hepatitis B virus positive, C+; hepatitis C virus positive, NL; normal liver, CH; chronic hepatitis, LF; liver fibrosis, LC; liver cirrhosis, TAE: transcatheter arterial embolisation, TACE; transcatheter arterial chemo-embolisation, RFA; radiofrequency ablation, PEIT; percutaneous ethanol injection therapy.

**Supplementary Table S2.** Fluorescence intensity increase in tumour tissues from freshly resected clinical hepatocellular carcinoma specimens from 0 to 30 min after spraying SPiDER-βGal.

The increase in fluorescence intensity in tumour from freshly resected clinical hepatocellular carcinoma specimens from 0 to 30 min after spraying SPiDER-βGal was analysed with different clinicopathological factors. No difference was observed among clinicopathological factors.

A two-tailed Mann-Whitney *U*-test was used. NL; normal liver, CH; chronic hepatitis, LF; liver fibrosis, LC; liver cirrhosis, HBV; hepatitis B virus positive, HCV; hepatitis C virus positive, non-B; hepatitis B virus negative, non-C; hepatitis C virus negative.
